# Simultaneous Fluorescent Recordings of Extracellular ATP and IntracellularCalcium in Mammalian Cells

**DOI:** 10.21769/BioProtoc.3242

**Published:** 2019-05-20

**Authors:** Nicholas Mikolajewicz, Svetlana V Komarova

**Affiliations:** 1 Faculty of Dentistry, McGill University, Montreal, Canada; 2 Shriners Hospital for Children–Canada, Montreal, Canada

**Keywords:** Fluorescence, Fura2, Extracellular ATP, Intracellular calcium, Luciferin, P2 receptors, Mammalian cells

## Abstract

Extracellular ATP is a potent signaling molecule that stimulates intracellular calcium responses through purinergic (P2) receptors in mammalian cells. While extracellular ATP and intracellular calcium can be measured separately, simultaneous monitoring can offer additional insights into P2 receptor physiology. This protocol takes advantage of the overlapping fluorescence spectra between the ATP-detection substrate luciferin and calcium indicator dye Fura2. Mammalian cells are loaded with Fura2-AM and live-cell recordings are acquired in the presence of a luciferin-luciferase imaging solution. This protocol allows to study stimulus-induced ATP release and directly relate changes in extracellular ATP concentration to observed calcium responses.

## Background

ATP is a potent extracellular signaling molecule that is released in response to a variety of stress-related stimuli, including mechanical stimulation or injury (Mikolajewicz *et al.*, 2018a). Extracellular ATP is an autocrine and paracrine signal that acts through a number of purinergic (P2) receptors, which consist of two sub-families, P2X and P2Y receptors, and are omnipresent in virtually all mammalian cells (Burnstock and Verkhratsky, 2009). P2X receptors consist of 7 subtypes (P2X_1-7_) of ligand-gated cation channels that permit calcium influx upon stimulation. P2Y receptors include 8 subtypes of G-protein coupled receptors (P2Y_1-2, 4, 6, 11-14_), several which can induce release of calcium from intracellular calcium stores through inositol triphosphate.

The luciferin-luciferase bioluminescence assay is the gold standard for measuring ATP concentrations (Seminario-Vidal *et al.*, 2009). In the presence of ATP, luciferase oxidizes luciferin and produces bioluminescent light proportionally to the amount of ATP present (Table1). While sensitive and robust, bioluminescence method is difficult to employ in microscopy applications and requires specialized equipment due to the low signal intensity. Luciferin is also a cell-impermeable fluorescent molecule, a property that was originally explored by Sorensen and Novak (2001) to develop a fluorescence method to measure extracellular ATP. This method is compatible with standard fluorescence microscopy and relies on the detection of a decrease in luciferin fluorescence emission intensity when it is depleted in the presence of ATP (Table 1).

**Table 1.**
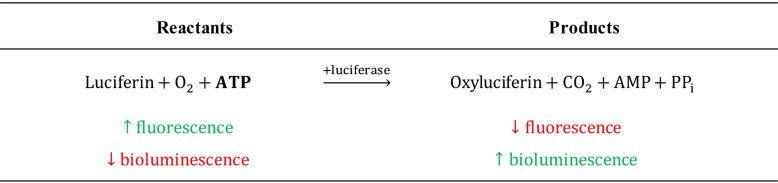
Bioluminescence and fluorescence properties of luciferin-luciferase reaction. In the presence of ATP, luciferin is oxidized by luciferase to generate oxyluciferin thereby resulting in production of bioluminescence and loss of fluorescence proportional to the concentration of ATP.

Calcium indicator dyes are used to monitor changes in cytosolic free calcium ([Ca^2+^]_i_) in live cells (Paredes *et al.*, 2008). While there is a wide selection of indicator dyes with varying properties (*e.g.*, affinity, brightness, spectral range), Fura2-acetoxymethyl ester (Fura2-AM) is among the most commonly used (Grynkiewicz *et al.*, 1985). When added to a cell culture, Fura2-AM accumulates intracellularly, where the acetoxymethyl ester group is removed by cellular esterases, producing an active dye entrapped within the cell. Fura2 is a ratiometric dye that changes its excitation spectrum upon binding Ca^2+^. When Ca^2+^-bound Fura2 is excited at 340 nm, emission at ~510 nm is proportional to [Ca^2+^]_i_. Conversely, when Ca^2+^-unbound Fura2 is excited at 380 nm, emission at ~510 nm is inversely proportional to [Ca^2+^]_i_. Thus, measuring fluorescence at a single emission of 510 nm, with alternating excitation with 340 and 380 nm, provides a ratiometric measure of [Ca^2+^]_i_ that is independent of dye loading and environmental artifacts.

**Figure 1.**
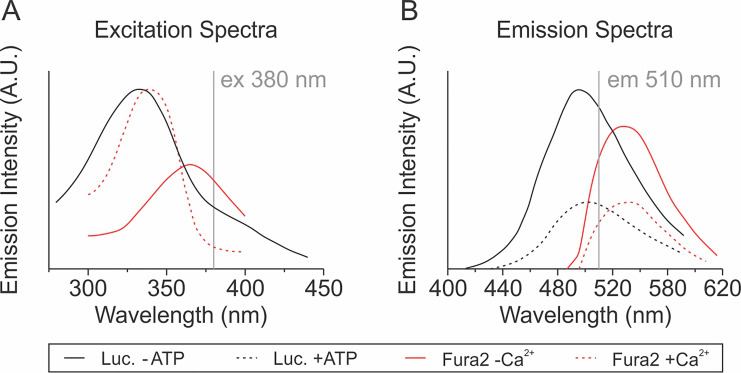
Fluorescent characteristics of D-luciferin and Fura2. A. Fluorescence excitation spectra for D-luciferin [Luc., em 550 nm (Goda *et al.*, 2015)] and Fura2 in the presence and absence of Ca^2+^ [em 500 nm (Grynkiewicz *et al.*, 1985)]. B. Fluorescence emission spectra of luciferin (Sorensen and Novak, 2001) and Fura2 (Grynkiewicz *et al.*, 1985) in the presence (+) and absence (-) of ATP and Ca^2+^, respectively.

Since the excitation and emission spectra of luciferin overlap with Fura2 (Figure 1), and the dyes are compartmentalized in the extracellular (luciferin) and intracellular (Fura2) spaces, this overlap in fluorescence spectra can be used to simultaneously measure extracellular ATP concentrations and changes in intracellular [Ca^2+^]_i_, as reported in our prior work (Mikolajewicz *et al.*, 2018b) and presented in detail below.

## Materials and Reagents

1. BMP2-transfected C2C12 myoblast cells (courtesy of Dr. M. Murshed, McGill University)
*Note: Any adherent mammalian cell line can be used for this protocol.*
2. Pipette tips
3. 35 mm glass-bottom dish (MatTek Corporation, catalog number: P35G-0-14-C)
4. 48-well glass-bottom culture plates (MatTek Corporation, catalog number: P48G-1.5-6-F)
5. Sterile disposable bottle top filters with polyethersulfone (PES) membrane, 0.2 μm pore size (Thermo Fisher Scientific, Nalgene Rapid-Flow, catalog number: 595-4520)
6. Pyruvate kinase from rabbit muscle (Sigma-Aldrich, catalog number: P9136)
7. Fura2-AM, cell permeant (Thermo Fisher Scientific, Invitrogen, catalog number: F1221)
8. D-Luciferin, potassium salt (Thermo Fisher Scientific, Invitrogen, catalog number: L2916)
9. Adenosine 5’-triphosphate magnesium salt, ATP (Sigma-Aldrich, catalog number: A9187)
10. Luciferase from Photinus pyralis (Sigma-Aldrich, catalog number: L9420)
11. Sodium chloride, NaCl (Thermo Fisher Scientific, Fisher Chemical, catalog number: S671-3)
12. Potassium chloride, KCl (Thermo Fisher Scientific, ACROS Organics, catalog number: 7447-40-7)
13. Sodium phosphate dibasic anhydrous, Na_2_HPO_4_ (Thermo Fisher Scientific, Fisher Chemical, catalog number: 7558-79-4)
14. Potassium dihydrogen phosphate, KH_2_PO_4_ (Thermo Fisher Scientific, Fisher Chemical, catalog number: 7758-11-4)
15. Magnesium chloride hexahydrate, MgCl_2_·6H_2_O (Thermo Fisher Scientific, Fisher Chemical, catalog number: 7786-30-3)
16. Calcium chloride dihydrate, CaCl_2_·2H_2_O (Thermo Fisher Scientific, Fisher Chemical, catalog number: 10035-04-8)
17. D-(+)-glucose (Sigma-Aldrich, catalog number: G7528)
18. HEPES, 1 M (Wisent Bioproducts, catalog number: 330-050-EL)
19. Phosphoenolpyruvic acid monopotassium salt (Sigma-Aldrich, catalog number: 860077)
20. Fura2 stock solution, 1,000x (see Recipes)
21. Luciferin stock solution, 5,000x (see Recipes)
22. Luciferase stock solution, 500x (see Recipes)
23. Physiological solution (PS), pH 7.4 (see Recipes)
24. Fura2-staining solution, 1x (see Recipes)
25. Luciferin imaging solution with luciferase (see Recipes)
26. Luciferin imaging solution without luciferase (see Recipes)
27. ATP solutions, 100x (see Recipes)
28. Phosphate-buffered saline (PBS), pH 7.4 (see Recipes)

## Equipment

1. Pipettes
2. Benchtop pH meter (Thermo Fisher Scientific, Mettler Toledo FE20 FiveEasy, catalog number: 10526655)
3. Levy counting chamber (VWR, Hausser Scientific, catalog number: 15170 208)
4. Benchtop Centrifuge (Precision, Durafuge 300)
5. Laminar flow biological hood
6. Water-jacketed CO_2_ incubator (Thermo Fisher Scientific, Forma Series II)
7. Vortex (Fisher Scientific, Vortex Genie 2, catalog number: 12-812)
8. Fluorescence inverted microscope (Nikon, Eclipse TE2000-U)
9. Fura2 Shemrock BrightLine Filter set (FURA2-A-000)
10. Oil immersion UV-corrected 40x lens (Nikon Plan Fluor 40x/1.30 Oil Objective)
11. UV Lamp excitation lamp (Sutter Ozone free 175 Watt xenon bulb Model # 0661176)
12. High speed wavelength switcher (Lambda DG-4, Quorum Technologies)

## Software

1. Excel (Microsoft)
2. Volocity (Improvision)
3. *Optional*: ImageJ (NIH)
*Note. ImageJ is freely accessible image analysis software that can be used as alternative to Volocity to define regions of interest (ROI) and extract data from image stacks. See Data Analysis section.*

## Procedure

- Fura2 loading
  1. 2-3 days prior to experiment, plate cells in uncoated 35 mm glass-bottom dish (or uncoated 48 mm glass-bottom culture plates) and allow cells to grow to sub-confluence in CO_2_ incubator (5% CO_2_, 37 °C).
  2. On the day of the experiment, aspirate medium and add 1 ml Fura2 staining solution (solution is at room temperature when added). Incubate cells at room temperature for 30 min in the dark.
  3. Aspirate Fura2-staining solution, and wash cells twice with physiological solution. Add imaging solution with luciferase (990 μl/35 ml dish or 297 μl/well in 48-well plate) and allow cells to acclimatize for 10 min at room temperature on the bench (in light-limiting conditions) prior to imaging.
- Instrument preparation
  1. Turn on the UV lamp to warm up 10-20 min prior to imaging.
  2. When ready to image, add drop of oil (Carl Zeiss^TM^ Immersol^TM^ Immersion Oil, Fisher Scientific, catalog number: 12-070-397) onto 40x objective lens and position glass bottom dish/plate on top of lens. Bring cells into focus and ensure cell and cell-free regions are visible within the field-of-view.
  3. Open imaging software (*e.g.*, Volocity) and enter the following imaging parameters:
**Sampling rate:** 2 timepoints per second
**Exposure time:** 50-200 ms
*Note: Find minimal exposure time required to obtain low-noise images. Longer exposures may result in photobleaching effects.*
**Excitation/emission:** 380 nm/510 nm
*Optional: If automated shutter is available (e.g., Lambda DG-4), close shutter between exposures to minimize photobleaching effects.*
- Imaging
  1. For all imaging trials, record 10-15 s of baseline, apply stimulation and record for additional 100-120 s. Stimulation can be mechanical, for example using a glass micropipette as described in our prior work (Mikolajewicz *et al.*, 2018b), or can be a biochemical stimulus carefully added at 1% volume at 100x of the desired final concentration.
*Note: For representative fluorescent images of Fura2-loaded cells bathed in luciferin imaging solution, refer to our prior work (Mikolajewicz et al., 2018b). Also see Sorensen and Novak, (2001) for images of cells bathed in luciferin-imaging solution.*
  2. To control for effects of drugs/solutions on D-luciferin signal, acquire recordings of cell-free wells containing luciferase-free imaging solution following stimulation with vehicle alone versus vehicle + drugs.
  3. To control for effects of drugs/solutions on luciferase activity, acquire recordings of cell-free wells containing luciferase-supplemented imaging solution following stimulation with ATP alone versus ATP + drugs.
  4. To control for effects of mechanical agitation resulting from the application of drugs, add vehicle (*e.g.*, physiological solution) to wells containing Fura2-loaded cells bathed in luciferase-supplemented imaging solution.
  5. To establish ATP calibration curve for luciferin recordings, carefully add 1% volume of 100x ATP solution (*e.g.*, 3 μl of 100 μM ATP added to 297 μl imaging solution in 48-well plate to achieve 1 μM ATP stimulation) into the well containing imaging solution, in presence and absence of luciferase (positive and negative controls shown in Figure 2).
*Notes:*
    - *Triplicate wells are recommended for each concentration.*
    - *Concentrations ranging from 0.1-1,000 μM ATP are recommended.*
    - *Calibration curve can be established in the absence or presence of fura2-loaded cells. The latter is recommended to better mimic experimental conditions and control for the influence of any cell-derived factors that may influence luciferase activity. Measuring Fura2-responses in the same wells also provides additional information about the range of ATP concentrations that Fura2-loaded cells will respond to.*
  6. The application protocols should be the same for experimental recordings, controls and calibration.
    **Figure 2.**
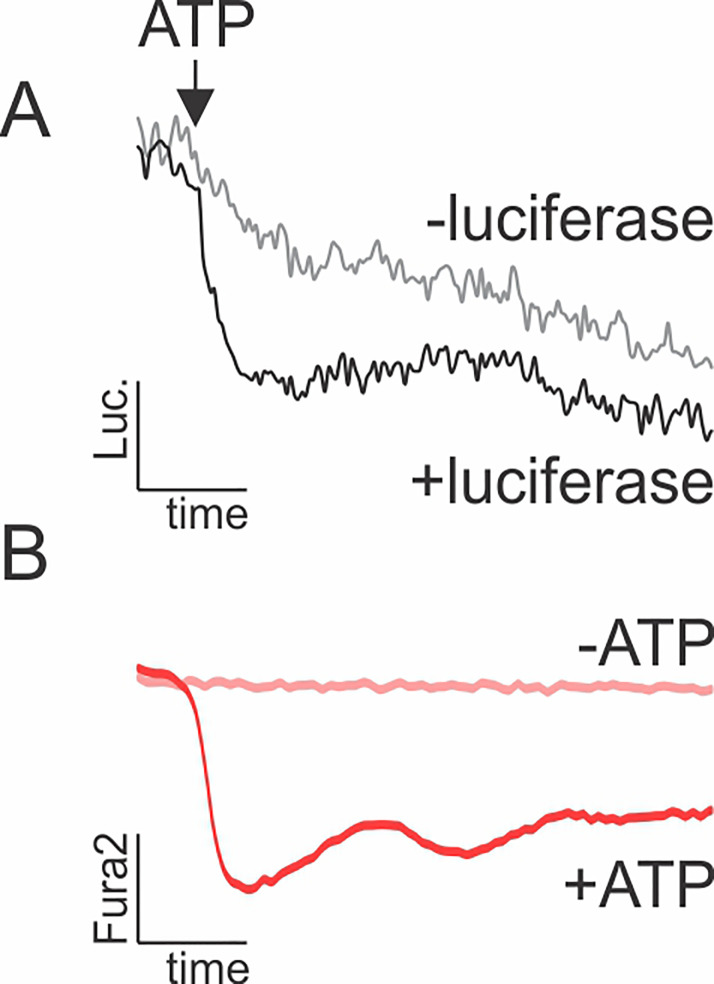
Effect of ATP on D-luciferin and Fura2 fluorescence (ex 380/em 510). A. Fluorescence of luciferin in the presence and absence of luciferase following addition of 1 μM ATP. B. Fluorescence of Fura2-loaded cells stimulated by 1 μM ATP (+ATP) or vehicle (-ATP).

## Data analysis

- Data extraction
  1. For each recorded image stack, define intracellular and extracellular regions of interest (ROI) manually and extract time-series data into a spreadsheet.
*Notes*:
    - *Intracellular ROIs (Fura2) are used for monitoring intracellular calcium responses and are manually selected to include the entire area of the cell.*
    - *
Extracellular ROIs (luciferin) are used to measure extracellular ATP concentrations and are selected ~15 µm from the cell’s edge. Smaller ROIs provide greater spatial resolution but are typically noisier. We recommend ROI selections of ~15^2^ um^2^. Avoid selecting extracellular ROIs immediately adjacent to the cell surface as these areas may exhibit “shadows” due to non-linear optics.
*
    - *To extract data in Volocity, draw an ROI using ROI tools and select multiple ROIs while holding ‘shift’ key. To extract tabular data from selections, select ‘Make Measurement Item’ from ‘Measurements’ menu, specify ‘All timepoints’ option and press ‘OK’. A new measurement item will appear in the Volocity library which can then be exported to in a tab-delimited format using ‘Export’ in the ‘file’ tab.*
    - *Optional: ImageJ is a freely accessible image analysis software which can be used for data extraction (as an alternative to Volocity). Online resources are available detailing how to select ROIs and extract data from image stacks in ImageJ.*
  2. Import luciferin and Fura2 data into separate Excel spreadsheets.
- ATP analysis
  1. To estimate extracellular ATP concentrations, determine the ATP calibration curve by calculating the absolute percentage change in average luciferin fluorescence $L_{i}^{j}$ pre and post stimulation:
    $${}_{luc}\left(\%\right)=\left|\frac{L_{post}^{\left(+luc\right)}}{L_{pre}^{\left(+luc\right)}}-\frac{L_{post}^{\left(-luc\right)}}{L_{pre}^{\left(-luc\right)}}\right|\times 100\%$$
where i specifies pre- or post-ATP application and j indicates the presence (+luc) or absence (-luc) of luciferase in the imaging solution.
*Notes*:
    - $L_{post}^{\left(-luc\right)}/L_{pre}^{\left(-luc\right)}\;$ *is the negative control.*
    - $L_{pre}^{j}$ *is the baseline signal (prior to stimulation) which can be either (1) the average signal intensity in the first 10 s of the recording or (2) the maximal intensity observed prior to stimulation.*
    - $L_{post}^{j}$ *is the post-stimulation signal which is typically taken as the average intensity 10-20 s post-stimulation.*
  2. Plot ∆${}_{luc}$ as a function of ATP concentration (log_10_ scale) and obtain equation for the line of best fit.
*Note: We used an exponential curve (∆*${}_{luc}=ae^{blog_{10}(\left[ATP\right])}$*) to fit the relationship between ATP concentration [ATP] (Molar) and* ∆${}_{luc}\left(\%\right)$*. Parameter estimates:* a=60 *and* b=0.55*, with a goodness of fit* $R^{2}=0.998$.
  3. Use ∆${}_{luc}\left(\%\right)$ from experimental conditions and relate to extracellular ATP concentration using calibration curve (Figure 3).
- Calcium analysis
  1. For Fura2 recordings, quantitative analysis is limited since only one excitation wavelength is recorded, however the presence or absence of a calcium response can be discerned and related to local ATP concentrations.
*
Note: If low extracellular ATP concentrations are expected (< 0.1 µM), a dose-dependency curve for Fura2 is recommended to determine the lowest ATP concentration that can stimulate a discernable response (Figure 3–red curve).
*
  2. Plot Fura2 recordings as a function of time and determine (*1*) the number of responding cells, *i.e.*, those that exhibit a > 4 standard deviation (STD) decline in Fura2 signal from baseline (Lopez-Ayon *et al.*, 2014) and (*2*) the total number of cells imaged in the field of view.
  3. Calculate the responding fraction of cells as $Responding\;fraction=\frac{responding\;cells}{total\;cells}$ (Figure 3).
    **Figure 3.**
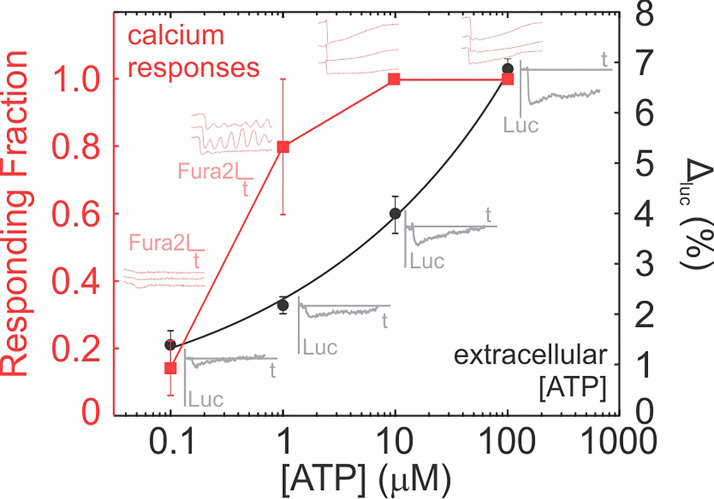
ATP dose-response curves for Fura2 and D-luciferin. Representative traces for intracellular Fura2 emission in individual Fura2-loaded cells (*light red traces*) and changes in extracellular luciferin (*gray traces*) are shown. Luc: luciferin emission (380 em/510 ex), Fura2: Fura2 emission (380 em/510 ex), t: time, Δluc: Percentage change in luciferin emission. Step-by-step analysis of the raw data used to generate this figure is provided as separate excel spreadsheets (fura2 analysis.xlsx and luciferin analysis.xlsx).

## Notes

1. If alternative imaging solution is used, ensure it is phenol red-free to avoid interference.
2. The protocol described here details how to image extracellular ATP and intracellular calcium simultaneously, however each can be done independently with appropriate amendments to the protocol.
3. Due to calcium-chelating properties of Fura2, Fura2-loaded cells will begin to lose viability over time. It is recommended that all cell imaging be completed within 30-60 min after Fura2-loading.
4. Ensure any treatments used in imaging experiments are assessed to determine whether they interfere with Fura2 or luciferin signal.
5. It may be necessary to adjust luciferin and luciferase concentrations for optimal results. Sorensen and Novak (2001) obtained satisfactory results using up to 1.67 mM D-luciferin and 25-188 μg/mL luciferase in a bicarbonate-free Ringer solution instead of the physiological solution described here (Sorensen and Novak, 2001).
6. A limitation of the current protocol is difficulty in achieving an instantaneously homogenous solution upon addition of drugs. The simplest solution is to plan for a larger sample size to negate noise that arises from inhomogeneous addition of solutions. Alternatively, a perfusion system may be adopted to deliver drugs, however, changes in ATP concentrations may be more difficult to detect due to the washout effect of the perfusion system. Nonetheless, in certain studies, such as those examining the effects of fluid flow on vascular calcium responses and ATP release, this may be a feasible alternative.

## Recipes

1. Fura2 stock solution, 1,000x
  1 μg/μl Fura2-AM in DMSO
  Store at -20 °C, protect from light
2. Luciferin stock solution, 5,000x
  30 mM D-luciferin in physiological solution
  Store at -80 °C, protect from light
3. Luciferase stock solution, 500x
  1 mg/ml firefly luciferase in physiological solution
  Store at -80 °C, protect from light
4. Physiological solution (PS), pH 7.4
  130 mM NaCl
  5 mM KCl
1 mM MgCl_2_
1 mM CaCl_2_
  10 mM glucose
  20 mM HEPES
  Sterilized by 0.2 μm vacuum filtration
  Store at 4 °C
5. Fura2-staining solution, 1x
  Fura2 stock solution (1:1,000 dilution)
  Physiological solution
  Prepare fresh, protect from light
6. Luciferin imaging solution with luciferase
  2 μl 30 mM D-luciferin stock solution (1:5,000 dilution)
  40 μl of 1 mg/ml firefly luciferase stock solution (1:250 dilution)
  10 ml physiological solution
  Prepare fresh, protect from light
7. Luciferin imaging solution without luciferase
  2 μl 30 mM D-luciferin stock solution (1:5,000 dilution)
  10 ml physiological solution
  Prepare fresh, protect from light
8. ATP solutions
  10 μM-100 mM ATP in luciferin imaging solution without luciferase (1x)
  Prepare fresh, protect from light
*Notes:*
  - *For ATP calibration curve, prepare 10 mM ATP solution and serially dilute in luciferin imaging solution to obtain lower concentrations (i.e., 1 in 10 dilution to obtain 1 mM ATP, etc.).*
  - *
ATP solutions often contain ADP (Mikolajewicz et al., 2019); we advise to prepare homogenous ATP stock solution by enzymatically converting contaminating ADP to ATP. This is accomplished by adding 5 μl 20 U/ml pyruvate kinase and 5 μl 100 μM phosphoenolpyruvate (PEP) into ATP stock solution, incubating for 30 min at 37 °C, then heat-inactivating for 5 min at 95 °C.
*
9. Phosphate-buffered saline (PBS), pH 7.4
  140 mM NaCl
  3 mM KCl
10 mM Na_2_HPO_4_
2 mM KH_2_PO_4_
  Sterilize by autoclaving, store at room temperature

## Supplementary Data

luciferin analysis.xlsx

## References

[1] Burnstock G. and Verkhratsky A.(2009). Evolutionary origins of the purinergic signalling system. Acta Physiol(Oxf) 195(4): 415-447. 19222398 10.1111/j.1748-1716.2009.01957.x

[2] Goda K., Hatta-Ohashi Y., Akiyoshi R., Sugiyama T., Sakai I., Takahashi T. and Suzuki H.(2015). Combining fluorescence and bioluminescence microscopy. Microsc Res Tech 78(8): 715-722. 26096873 10.1002/jemt.22529 PMC4745033

[3] Grynkiewicz G., Poenie M. and Tsien R. Y.(1985). A new generation of Ca^2+^ indicators with greatly improved fluorescence properties . J Biol Chem 260(6): 3440-3450. 3838314

[4] Lopez-Ayon G. M., Liu H. Y., Xing S., Maria O. M., LeDue J. M., Bourque H., Grutter P. and Komarova S. V.(2014). Local membrane deformation and micro-injury lead to qualitatively different responses in osteoblasts. F1000Res 3: 162. 25254108 10.12688/f1000research.4448.1 PMC4168753

[5] Mikolajewicz N., Mohammed A., Morris M. and Komarova S. V.(2018). Mechanically stimulated ATP release from mammalian cells: systematic review and meta-analysis. J Cell Sci 131(22). 10.1242/jcs.223354 30333142

[6] Mikolajewicz N., Zimmermann E. A., Willie B. M. and Komarova S. V.(2018). Mechanically stimulated ATP release from murine bone cells is regulated by a balance of injury and repair. Elife 7: e37812. 30324907 10.7554/eLife.37812 PMC6205812

[7] Mikolajewicz N., Sehayek S., Wiseman P. W. and Komarova S. V.(2019). Transmission of mechanical information by purinergic signaling. Biophys J 116(10): 9654. 10.1016/j.bpj.2019.04.012 PMC6531813 31053261

[8] Paredes R. M., Etzler J. C., Watts L. T., Zheng W. and Lechleiter J. D.(2008). Chemical calcium indicators. Methods 46(3): 143-151. 18929663 10.1016/j.ymeth.2008.09.025 PMC2666335

[9] Seminario-Vidal L., Lazarowski E. R., and Okada S. F.(2009). Assessment of extracellular ATP concentrations. Methods Mol Biol 574: 25-36. 19685297 10.1007/978-1-60327-321-3_3

[10] Sorensen C. E. and Novak I.(2001). Visualization of ATP release in pancreatic acini in response to cholinergic stimulus. Use of fluorescent probes and confocal microscopy. J Biol Chem 276(35): 32925-32932. 11387334 10.1074/jbc.M103313200

